# Idelalisib and caffeine reduce suppression of T cell responses mediated by activated chronic lymphocytic leukemia cells

**DOI:** 10.1371/journal.pone.0172858

**Published:** 2017-03-03

**Authors:** Barry D. Hock, Sean A. MacPherson, Judith L. McKenzie

**Affiliations:** 1 Haematology Research Group, Christchurch Hospital, Christchurch, New Zealand; 2 Pathology Department, University of Otago, Christchurch, New Zealand; 3 Haematology Department, Christchurch Hospital, Christchurch, New Zealand; University of Manitoba, CANADA

## Abstract

Chronic lymphocytic leukemia (CLL) is associated with T cell dysfunction. Activated CLL cells are found within the lymphoid tumor micro-environment and overcoming immuno-suppression induced by these cells may improve anti-CLL immune responses. However, the mechanisms by which activated CLL cells inhibit T cell responses, and reagents targeting such mechanisms have not been identified. Here we demonstrate that the ability of *in vitro* activated CLL cells to suppress T cell proliferation is not reversed by the presence of ecto-nuclease inhibitors or blockade of IL-10, PD-1 and CTLA-4 pathways. Caffeine is both an adenosine receptor antagonist and a phosphatidylinositol-3-kinase, p110δ (PI3Kδ) inhibitor and, at physiologically relevant levels, significantly reversed suppression. Significant reversal of suppression was also observed with the PI3Kδ specific inhibitor Idelalisib but not with adenosine receptor specific antagonists. Furthermore, addition of caffeine or Idelalisib to activated CLL cells significantly inhibited phosphorylation of AKT, a downstream kinase of PI3K, but did not affect CLL viability. These results suggest that caffeine, in common with Idelalisib, reduces the immuno-suppressive activity of activated CLL cells by inhibiting PI3Kδ. These findings raise the possibility that these compounds may provide a useful therapeutic adjunct by reducing immuno-suppression within the tumor micro-environment of CLL.

## Introduction

B-cell chronic lymphocytic leukemia (CLL) is associated with a profound immuno-suppression which results in both impaired anti-tumor responses and increased susceptibility to infection [1]. T-cells are central to the development of effective immune responses and studies on both the T cells circulating in CLL patients and those present in CLL-T cell co-cultures provide strong evidence that CLL cells can impair T cell function [2–8]. Understanding the mechanisms underlying this process is a key step in developing new therapies that can reduce immune dysfunction and thereby improve anti-tumor responses [2, 3].

It has become recognised that, within lymphoid tissue, the complex interaction of CLL cells with the tumor micro-environment (TME) provides signals necessary to sustain tumor progression and immune evasion [2, 3, 9]. Within the so-called pseudo follicles of the TME activated CLL cells are found in close contact with activated T cells, and it is thought this interaction is critical for CLL progression [10–12]. However, it is unclear how activated CLL cells suppress anti-tumor responses.

Studies to date on the immunosuppressive capacity of CLL cells have primarily utilised non-activated, circulating CLL populations. Data both from these studies and those using similarly immunosuppressive regulatory B cells (Bregs) [1, 13] suggest a number of potential pathways by which CLL cells may deliver inhibitory signals. These include expression of inhibitory ligands such as CD274 and CD276 [6, 10], release of cytokines such as IL10 [14] and enzymatic generation of adenosine through the activity of the ecto-enzymes CD38, CD39 and CD73 [15–18]. The B cell receptor (BCR) signalling pathway is central to CLL activation within the TME. Inhibitors such as Idelalisib, which target the phosphatidylinositol-3-kinase (PI3K) isoform p110δ (PI3K δ) downstream of the BCR, have numerous effects on CLL progression [19–21]. However, their effect on CLL mediated suppression is unknown. The methylxanthine caffeine is potentially a modulator of CLL mediated suppression due to its activity as both an adenosine receptor antagonist and a PI3K inhibitor [22–24]. However, its effect on CLL cells has not been evaluated.

We have previously utilised a CLL + activated T cell co-culture system as an *in vitro* model of the pseudo follicles of the TME and demonstrated that the activated CLL cells generated are capable of suppressing polyclonal T cell responses [8, 25]. The pathways by which these activated CLL induce suppression are currently unknown and are investigated in this study using a range of agonists and antagonists that target potential pathways. We demonstrate that both caffeine and Idelalisib reverse the suppressive activity of activated CLL cells.

## Materials and methods

### Reagents

Monoclonal antibodies used for phenotypic analysis were CD276-FITC (B7-H3) from R&D Systems. CD274-FITC (B7-H1, PDL-1), CD80-PE, CD86-FITC, CD19-FITC, CD19-PE, CD3-PE-Cy7 and PE-anti Akt (S473) were obtained from BD Biosciences.

Blocking antibodies and recombinant proteins used in proliferation assays were CD80 (clone 2D10), CD152 (CTLA-4, clone L3D10) from BioLegend. CD279 (PD1, clone J116), anti-human CD274 (PDL-1, clone MIH1) and mouse IgG1 control were obtained from eBioscience. Anti-IL-10 (clone 23738), recombinant B7-1/CD80 and recombinant CTLA-4/CD152 were from R&D Systems. All blocking antibodies and recombinant proteins were used at 10ug/ml final concentration.

Ecto-nuclease inhibitors used (abbreviation, final concentrations) were CD38 inhibitor, Kuromanin (25um); CD73 inhibitor, α,β-Methyleneadenosine 5’-diphosphate sodium salt (APCP,100uM) and CD39 inhibitor, ARL67156, (ARL, 10uM) from Sigma. The adenosine receptor antagonists used were (receptor specificity, final concentrations) CGS1593 (A1+A2A+A2B+A3, 500nM), PSB36 (A1, 50nM), PSB1115 (A2B, 500nM) and PSB10 (A3, 50nM) from R&D Systems, and SCH58261 (A2A, 100nM) was obtained from Sigma.

The ryanodine receptor inhibitor 4-chloro-3-methyphenol (4CMP, 200uM) and caffeine (200uM) were obtained from Sigma, and the PI3K inhibitor Idelalisib (100nM) was from Selleck Chemicals. Functional grade anti-Human IgM + IgG (10ug/ml) was from eBioscience.

### Isolation of peripheral blood mononuclear cells and activation of CLL cells

This study was reviewed and approved by the Upper South A regional Ethics Committee, NZ (Ethics reference URA/08/08/050). Blood was collected from normal donors or CLL patients following written informed consent using a consent form approved by the ethics committee. Signed consent forms were filed and stored in the Haematology Department, Christchurch Hospital. Untreated CLL patients with white cell counts >30, were recruited through the Haematology Department, Christchurch Hospital. Peripheral blood mononuclear cells (PBMC) were prepared from normal donor blood for use as responders in functional assays and were labelled with carboxyfluorescein succinimidyl ester (CFSE) as previously described [8, 26]. For some experiments CFSE labelled PBMC were depleted of monocytes using CD14 mAb and immuno-magnetic Dynabeads [26].

If required, CLL cells were enriched from the patient PBMC by negative selection using magnetic beads as described previously [8]. All CLL preparations contained >95% CLL cells (CD19^+^, CD5^+^). Cells were stored in liquid N_2_ until use.

CLL cells were activated as previously described [8]. Briefly, CLL cells were cultured with normal donor PBMC in the presence of CD3/CD28 antibodies for 3 days, and the CLL^Act^ purified using CD19 positive selection. Purified CLL^Act^ were >95% pure as assessed by flow cytometry.

For phenotypic analysis cultures were double-labelled with monoclonal antibodies to CD19 and either CD38, CD80, CD86, B7H-1 or B7H-3 followed by analysis of CD19+ cells.

For PI3K analysis CLL^Act^ were cultured with or without inhibitors for 1 hr at 37C, followed by activation with anti-IgM+IgG for 15 mins. Following labelling with CD19-FITC, cells were fixed using Fixation Medium A as recommended by the manufacturer (Caltag). Cells were then permeabilised with cold methanol (30min) and then stained with antibody specific for AKT phosphorylated at Serine 473 (PE-anti Akt (S473)) as recommended by the manufacturers (BD Biosciences).

Cells were analysed on a Beckman Coulter FC500 MPL flow cytometer, and results are expressed as mean fluorescence intensity (MFI).

### Functional assays

CD3/CD28 induced T cell proliferation assays were performed in plates coated with CD3 mAb as previously described [8]. Briefly, CFSE labelled PBMC (CFSE^+^ PBMC) were cultured with or without CLL cells in the presence of CD28. After 72h incubation supernatants were harvested and frozen whilst cells were labelled with CD3-PE and analysed by flow cytometry. Quantification of T cell proliferation based on the flow cytometric data was performed as follows (S1 Fig) using CXP analysis software (Beckman Coulter). The viable cells were gated using plots of forward versus side scatter and their associated fluorescence then shown as dot plots of CFSE-FITC versus CD3-PE. Following gating on the CD3^+^CFSE^+^ T cell population the CFSE associated fluorescence was shown as a histogram. Within these histograms each round of T cell division is associated with serial dilution of the initial T cell fluorescence and the percentage of T cells that had undergone ≥ 3 divisions was determined. Percentages were normalised relative to the percentage observed in cultures containing PBMC and CLL cells alone, which represents baseline suppression. These normalised values were defined as relative proliferation (Relative proliferation (%) = percentage ≥ 3 divisions / percentage ≥ 3 divisions in PBMC + CLL cultures x 100). Unless otherwise indicated activated CLL cells were added at the start of the proliferation assay (t = 0). In one set of experiments the CLL cells were added to cultures 24h after the initiation of t cell activation.

### Inhibition studies

To analyse the effect of various agents on the suppressive capacity of CLL^Act^, inhibitors were added to both the PBMC alone as a control for any possible direct effect on T cells, and to the PBMC/CLL co-cultures at the start of the assay. Proliferation was analysed at 72 hours as above. Carrier only was used as negative controls. None of the inhibitors utilized in this study, including caffeine and Idelalisib significantly modulated the proliferative responses of PBMC cultured alone (S2 Fig).

Apoptosis was analysed by labelling co-cultures with Annexin V- PE at 48 hrs, and gating on either CLL cells (CFSE negative) or PBMC (CFSE positive). The proportion of Annexin V positive cells in the cultures containing inhibitor was normalised to the co-cultures containing PBMC, CLL and carrier alone.

### Statistical analysis

Statistical analysis was performed using GraphPad Prism 6. (GraphPad Software Inc). Statistical analysis of raw data was performed using a paired *t* test. P<0.05 was considered significant. *P<0.05;**P<0.01;***P<0.001;****P<0.0001

## Results

### Expression and function of B7 family molecules

**Fig 1:**
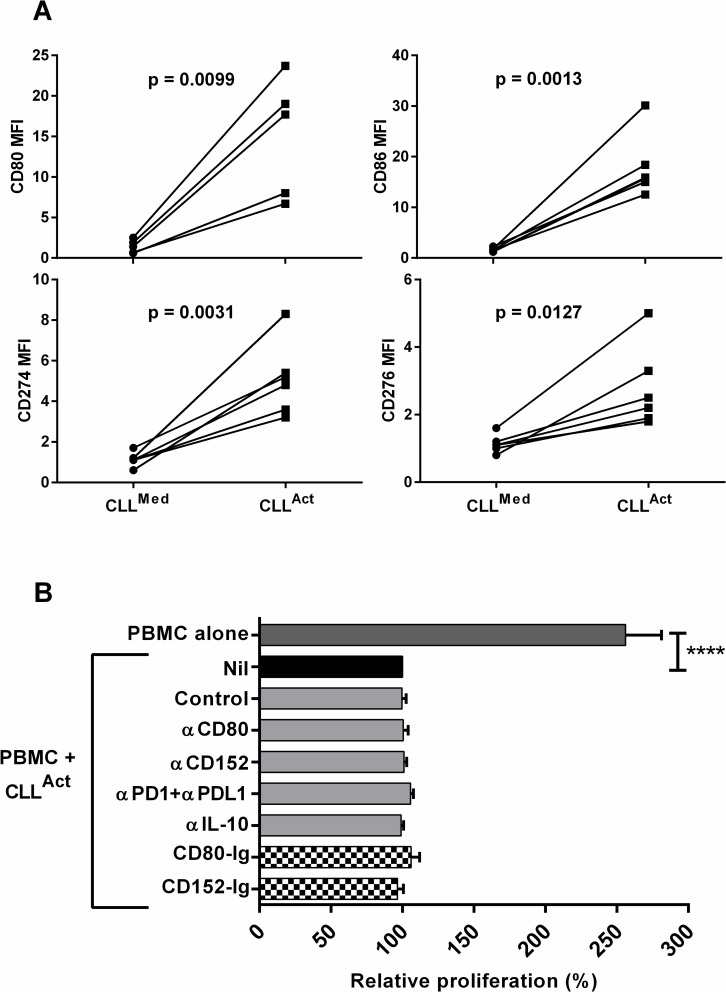
Expression and function of B7 family members. Purified CLL cells were cultured 72 hr either alone (CLL^Med^) or with allogeneic PBMC + CD3/CD28 (CLL^Act^). CLL were then analysed by flow cytometry or purified and utilized in proliferation assays. A) Flow cytometric analysis of antigen expression. Data are shown as scatterplots of the MFI of each molecule on CLL cells obtained from 5–6 individual CLL patients. Differences between CLL^Med^ and CLL^Act^ were analysed by paired *t*-test with respective *p* values reported in each plot. B) CFSE-PBMC were cultured with CD3/CD28 and the indicated combinations of media alone, purified CLL^Act^ and inhibitors (blocking antibodies (α), recombinant proteins (Ig) or controls) for 72 hours prior to analysis of proliferation. Data obtained from separate experiments with blocking antibodies (n = 4) and recombinant proteins (n = 3) were normalised relative to the respective cultures containing CLL^Act^ and control only (defined as 100%) and shown as relative proliferation (mean % ± SEM).

The co-culture of CLL cells with CD3-CD28 stimulated PBMC generates activated CLL (CLL^Act^) with the ability to suppress T cell responses [8]. Expression of B7 family members by CLL cells has been implicated in their immune-modulatory capabilities. Analysis of B7 family member expression by CLL^Act^ demonstrated that, relative to CLL cells cultured in media alone, levels of CD80, CD86, CD274 (PDL-1) and CD276 (B7H3) were all significantly increased (Fig 1A). The functional importance of these molecules in CLL^Act^ mediated suppression was analysed using both blocking antibodies and recombinant ligands (Fig 1B). CD274 (PDL-1) can deliver a T cell inhibitory signal by binding to either CD279 (PD-1) or CD80 [27]. The combined addition of blocking mAb specific for CD274 and CD279 had no significant effect on suppressive capacity and a similar lack of effect was observed using an anti-CD80 mAb (Fig 1B). CD80-Ig which is reported to be a more efficacious agent for overcoming CD274 mediated suppression [28] similarly had no significant effect. Addition of a CTLA-4 (CD152) specific blocking mAb or CTLA-4-Ig had no significant effect on suppression. IL-10 release has been implicated in the suppressive capacity of normal and malignant B cells. As we reported previously we did not observed increased IL-10 release by CLL^Act^ and, in keeping with those findings, addition of blocking IL-10 mAb did not reduce suppression induced by CLL^Act^ (Fig 1B).

### Effect of monocyte depletion and delayed CLL^Act^ addition

**Fig 2:**
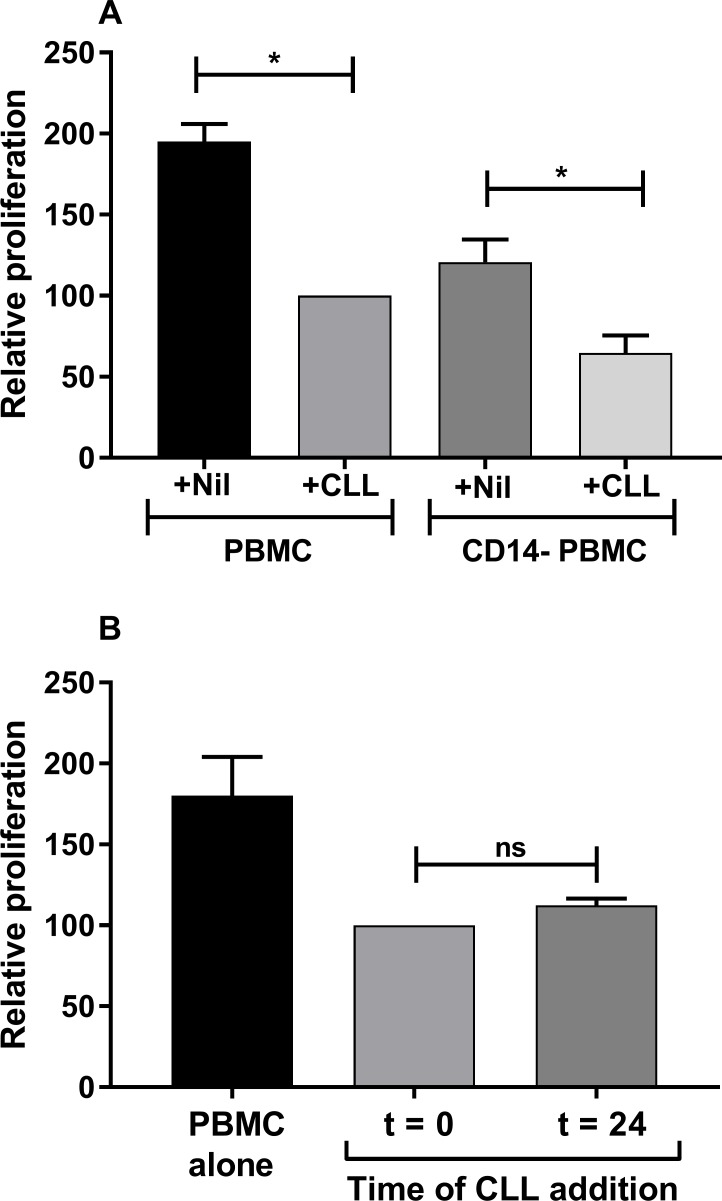
Effect of monocyte depletion and delayed CLL^Act^ addition on suppression. CFSE labelled responders were cultured with CD3/CD28 in the presence or absence of CLL^Act^ prior to analysis of proliferation. All data were normalised relative to cultures containing PBMC + CLL^Act^ added at T = 0 (defined as 100%) and shown as relative proliferation (mean ± SEM). (A) Relative proliferation observed in cultures using CFSE-PBMC responders that were either untreated or CD14 depleted prior to assay. Either nil or CLL^Act^ were added at T = 0. Data are from 4 separate experiments (B) Relative proliferation observed following culture of CFSE-PBMC either alone or in the presence of CLL^Act^ added at T = 0 or T = 24h. Data are from 4 separate experiments.

The requirements for CLL mediated suppression were further investigated. CLL cells may suppress indirectly by inducing monocytoid myeloid derived suppressor cells (MDSC) [29]. The effect of depleting monocytes from the responder PBMC was therefore analysed (Fig 2A). As expected CD14 depleted PBMC (CD14^-^PBMC) had reduced baseline proliferation compared to PBMC. Addition of CLL^Act^ induced significant suppression in both responder types and the degree of suppression was similar using CD14^-^PBMC and PBMC responders (median reduction in proliferation = 52% versus 53%).

T cells become committed to proliferation during the 1^st^ 24h of activation [30, 31]. The ability of CLL^Act^ to suppress if added later (T = 24h) to the proliferation assay was analysed (Fig 2B). Delayed addition of CLL^Act^ did not significantly alter the level of observed suppression. This suggests CLL^Act^ cells can suppress T cells that already have received initial activation signals.

### Effect of ecto-enzyme and adenosine receptor inhibitors

**Fig 3:**
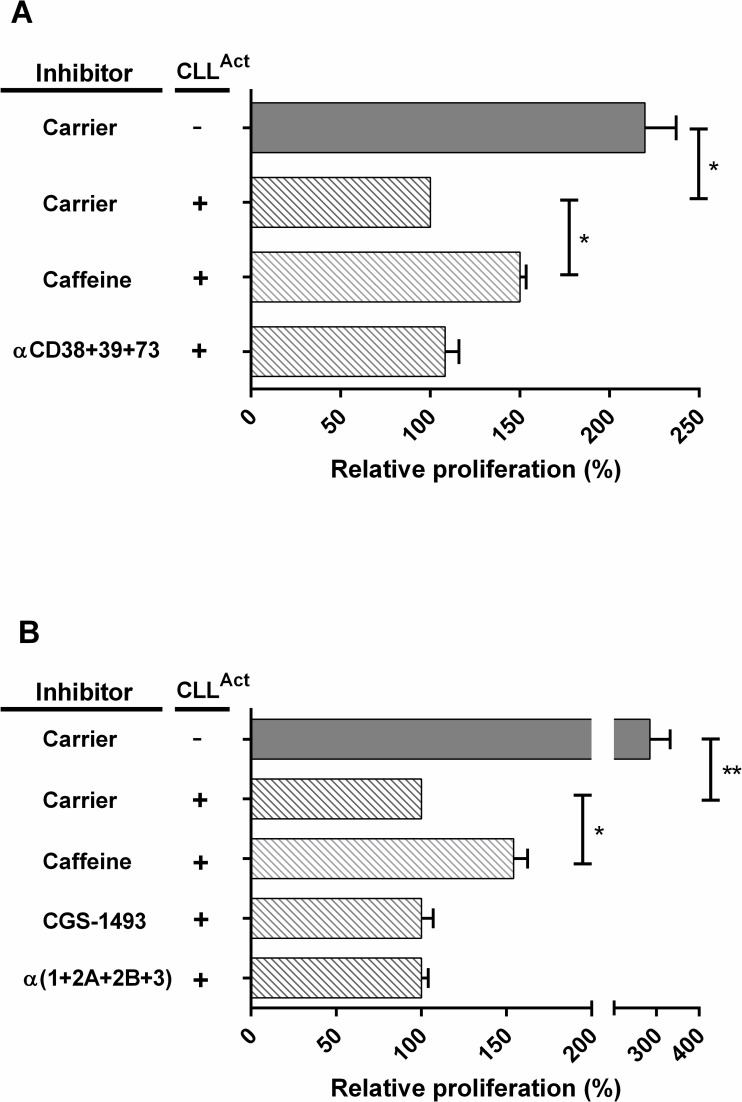
Effect of enzyme and adenosine receptor inhibitors on suppression mediated by CLL^Act^. Proliferation in the presence of inhibitors was normalised relative to cultures containing CLL^Act^ and carrier alone (defined as100%) and pooled data shown as relative proliferation (mean % ± SEM). (A) Relative proliferation observed following culture (n = 4 experiments) in the presence of carrier only control, caffeine or a combination of endonuclease inhibitors specific for CD38, CD39 and CD72. (B) Relative proliferation observed following culture (n = 6 experiments) in the presence of either carrier only control, caffeine, the adenosine receptor antagonist (CGS-1493) or a combination of antagonists specific for all adenosine receptor subtypes (1, 2A, 2B, 3).

The activity of the ecto-enzymes CD38, CD39 and CD73 and the subsequent binding of their major product adenosine to the adenosine receptor (ADR) subtypes represent potential suppressive mechanisms. Addition of a combination of inhibitors specific for the ecto-enzymes CD38, CD39 and CD73 did not significantly reverse suppression (Fig 3A). Next we added ADR antagonists specific for 1 or all 4 ADR subtypes (Fig 3B). Addition of caffeine which is an antagonist of all ADR subtypes induced partial but significant reversal of suppression. However, addition of the caffeine analog CGS 15943 which is also an antagonist of all ADR subtypes did not have a significant effect. Similarly, addition of ADR subtype specific antagonists, either alone (data not shown), or combined, did not reverse suppression.

### Effect of PI3K inhibition

Caffeine has other modes of action in addition to being an antagonist of adenosine receptors.

**Fig 4:**
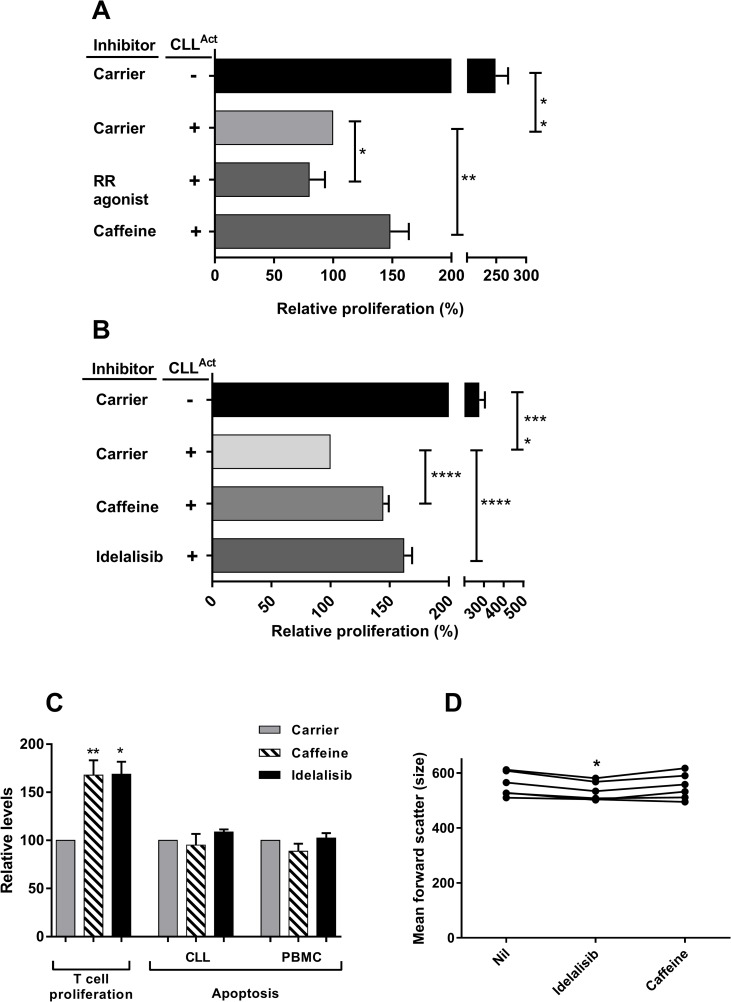
Effect of Ryanodine Receptor agonist and PI3K inhibitors on suppression induced by CLL^Act^. CFSE-PBMC were cultured with CD3/CD28 in the presence or absence of CLL^Act^ with or without the addition of caffeine, ryanodine receptor agonist 4CMP or the PI3Kδ inhibitor Idelalisib. Flow cytometric analysis was then performed to analyse apoptosis at 48h and proliferation and cell size at 72h. All data were normalised relative to cultures containing PBMC + CLL^Act^ and carrier only control (defined as 100%). A) Proliferation in the absence and presence of caffeine or 4CMP was analysed and normalized data from 5 experiments shown as relative proliferation (mean ± SEM) at 72 hrs. B) Proliferation in the absence and presence of caffeine or Idelalisib was analysed and normalized data from 11 experiments shown as relative proliferation (mean ± SEM) at 72 hrs. C) Bar graph of the relative T cell proliferation and relative apoptosis of the PBMC and CLL populations observed in activated co-cultures. Co-cultures were performed in the presence or absence of caffeine and Idelalisib. Normalised data from individual experiments (n = 4) are shown as mean ± SEM. D) Scatter plot of the size of the CLL cells present at the end of co-culture. Data from 6 separate experiments are shown as plots of mean forward scatter.

These activities include activation of ryanodine sensitive calcium channels and inhibition of PI3Kδ. Therefore, the effect of 4CMP, a ryanodine receptor agonist and Idelalisib, a PI3Kδ specific inhibitor, on CLL^Act^ mediated suppression was analysed (Fig 4). Addition of 4CMP resulted in a small, but significant increase in suppression whilst caffeine in the same experiments significantly reduced suppression (Fig 4A). However, addition of Idelalisib (100 nM), in common with caffeine, induced partial but significant reversal of suppression (Fig 4B). Neither caffeine nor Idelalisib affected the baseline proliferation of PBMC activated in the absence of activated CLL (S2 Fig). Next we investigated whether changes in the levels of apoptosis played a role in the effect of caffeine and Idelalisib (Fig 4C). In our previous study we reported that, compared to cells cultured alone, co-culture of PBMC and CLL in the suppression assay results in significantly lower CLL apoptosis but does not alter the alter levels of PBMC apoptosis [8]. Although the additional presence of caffeine and Idelalisib in the suppression assay significantly increased T cell proliferation there was no significant change in the levels of apoptosis in either the CLL or PBMC populations present in these co-cultures. PI3K inhibition can reduce cell size and therefore the size of the activated CLL cells following co-culture was analysed. The presence of caffeine did not significantly alter cell size whilst the presence of Idelalisib resulted in a small but significant reduction (Fig 4D).

**Fig 5:**
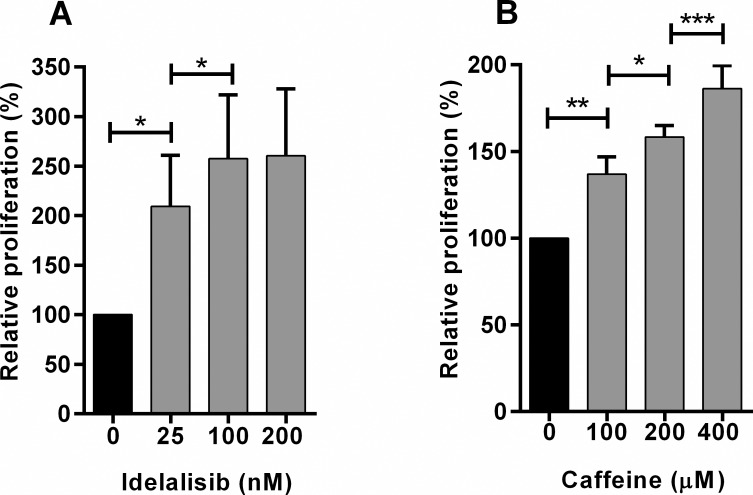
Dose dependent effects of caffeine and Idelalisib on suppression induced by CLL^Act^. CFSE^+^PBMC were cultured in the presence of CLL^Act^ and increasing concentrations of caffeine or idelalisib, prior to analysis of proliferation. All data were normalised relative to cultures containing PBMC + CLL^Act^ and carrier only control (defined as 100%) and shown as relative proliferation (mean ± SEM). A) Relative proliferation in the presence of increasing concentrations of Idelalisib (n = 3 experiments). B) Relative proliferation in the presence of increasing concentrations of Caffeine (n = 5 experiments).

In order to determine the concentration dependence of caffeine and Idelalisib mediated effects, titrations of these reagents were added to the suppression assay. Idelalisib had a significant effect at concentrations of 25, 100 and 200 nM although the magnitude of the response was significantly lower at 25 nm (Fig 5A). Caffeine had a significant effect on suppression at concentrations of 100, 200 and 400 μM (Fig 5B). Although caffeine had a significantly greater effect when the concentration was increased from 200 μM to 400 μM, this higher level is considered toxic *in vivo* and induced a small but significant reduction in the proliferation of PBMC cultured in the absence of CLL^Act^ (data not shown). Caffeine at this concentration was therefore not used further in this study.

**Fig 6:**
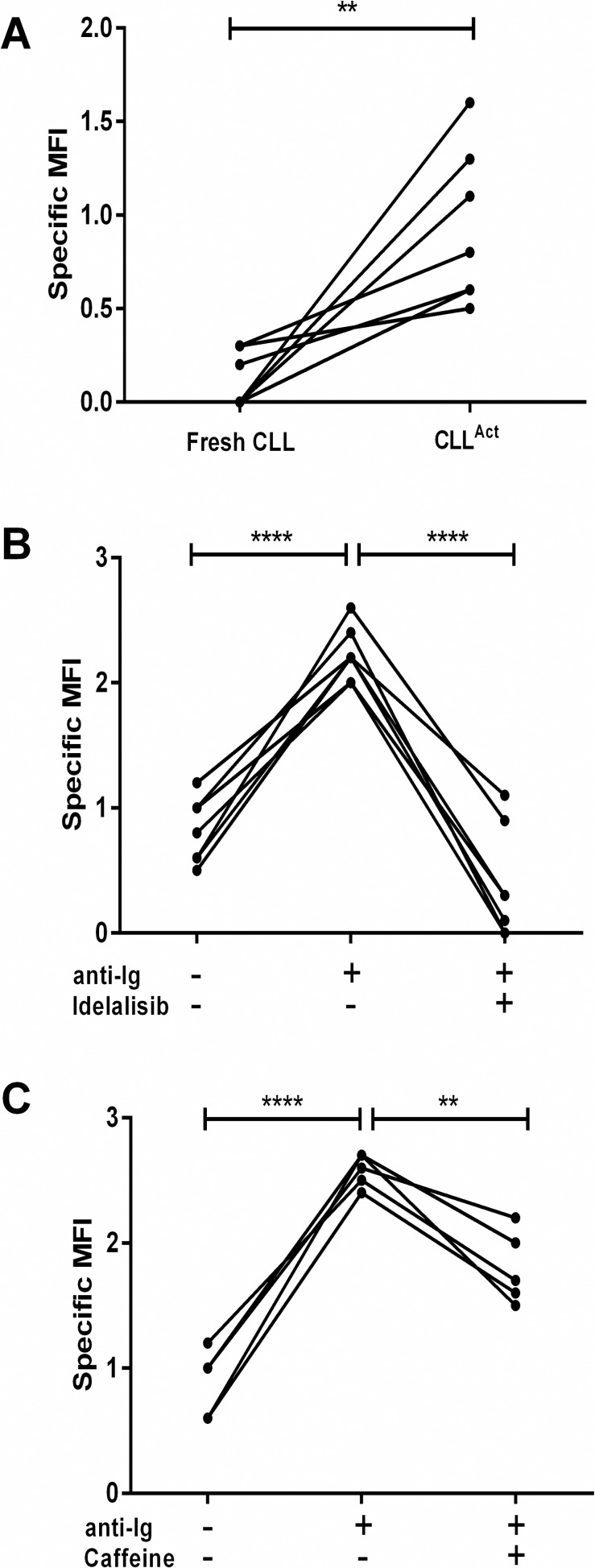
Effect of Caffeine and Idelalisib on pAkt (S473). Expression of pAKT by CLL cells was analysed by flow cytometry either prior to or following stimulation (15 min) with anti-Ig. (A) CLL cells (n = 7) were analysed before (CLL) or after (CLL^Act^) activation of cells by 72h co-culture with activated PBMC. (B, C) CLL^Act^ were cultured for 1 hr in the presence or absence of either (B) Idelalisib (n = 7) or (C) caffeine (n = 5) prior to stimulation with anti-Ig for 15 mins and analysis of pAkt (S473). Data from analysis of CLL cells from 7 different patients are shown as a scatter plot of specific MFI.

The level of PI3K activity in CLL^Act^ and the ability of caffeine and Idelalisib to inhibit this activity was then analysed (Fig 6). PI3K activity results in the activation and phosphorylation of the serine-threonine kinase AKT and the levels of phosphorylated AKT (pAKT) were therefore analysed by flow cytometry. CLL cells analysed pre-culture had low levels of pAKT. However CLL^Act^ generated by co-culture with CD3-CD28 stimulated PBMC had significantly higher levels of pAKT. Stimulation of circulating CLL cells by crosslinking the BCR is known to induce a rapid increase in pAKT [32]. BCR crosslinking of CLL^Act^ similarly induced a significant increase in pAKT. However crosslinking in the presence of Idelalisib or caffeine resulted in significantly lower levels of pAKT.

## Discussion

Within the TME activated CLL cells are found in close contact with activated T cells and this interaction is thought to play a critical role in CLL pathogenesis [2, 3, 9–11]. There are two disparate aspects to this interaction as, although the CLL cells require signals from the T cells for their activation and proliferation, they must also suppress T cell anti-tumor activity in order to escape elimination [2]. In this study, CLL cells activated by co-culture with activated T cells are used as an *in vitro* model to analyse the immunosuppressive mechanisms utilised by activated CLL populations [8, 25]. Identifying such mechanisms may allow development of blocking therapies that promote re-instatement of immune-surveillance [2, 3].

Studies using circulating CLL populations have clearly demonstrated that they can induce T cell dysfunction and, together with data from a murine model of CLL, have implicated molecules such as CD274, CD276, CTLA-4 and IL10 in this process [6, 10, 14, 33, 34]. However, in the current study the addition of a range of reagents targeting these pathways failed to reduce the observed suppression. This may reflect differences in the immune-regulatory mechanisms utilised by circulating and activated CLL cells. It is well established that such populations differ markedly with respect, not only to gene and protein expression profiles [11, 35, 36], but also their immunomodulatory capacity [8, 37]. In the current study, CLL activation induced increased levels of both costimulatory (CD80, CD86) and co-inhibitory (CD274, CD276) molecules suggesting a capacity to either stimulate or suppress T cell responses. This is consistent with data showing that both activated CLL cells and activated normal B cells are stimulatory in assays with relatively low levels of baseline T cell proliferation and suppressive in assays using polyclonally activated T cells [8, 38, 39]. Recent studies have suggested that the differentiation/activation status of T cells may modulate their response to populations such as CLL cells and Tregs [40, 41]. Our finding that activated CLL cells can suppress T cells that have already been exposed to activation signals suggests they do not need to be continuously present in order to suppress T cell responses.

An issue with *in vitro* immunosuppression assays is how to demonstrate that observed suppression is not merely due to the increased number of cells increasing media depletion and/or sterically reducing T cell access to stimulatory CD3 mAb. We have previously addressed this by examining different suppressor:responder ratios and showing a lack of suppression upon addition of non-activated CLL cells [8]. Our finding that delaying addition of activated CLL by 24h still results in a similar level of suppression provides additional evidence that the suppression is not due solely to an increased number of cells in the cultures. If this was the case, delayed addition of the activated CLL cells would be expected to reduce their suppressive effect, as exposure to increased numbers of “nutrient depleting” cells is effectively reduced by one third. Additionally with delayed addition, the CD3 mAb is not exposed to potential steric hindrance during the critical 1^st^ 24h of activation [30, 31]. Therefore, if steric hindrance was a factor, delayed addition would result in a reduced rather than unchanged level of suppression.

CLL cells have many features in common with immunosuppressive Bregs [1, 13]. One of the immunosuppressive mechanisms utilised by both of these cells is the ecto-enzymatic generation of immunosuppressive adenosine via the CD39/CD73 or CD38/CD203a/CD73 pathways [15–18]. Activated CLL cells within the pseudo follicles strongly express CD38, CD39 and CD73 and can also generate adenosine suggesting a role for these pathways in CLL^Act^ mediated suppression [11, 18]. Caffeine was initially utilised in this study because of its activity as an ADR antagonist [22, 42]. Although it partially reversed the suppression, the ineffectiveness of both ecto-enzyme inhibitors and ADR specific antagonists demonstrated that this effect did not directly involve this pathway. Additional effects mediated by low concentrations of caffeine include activation of ryanodine receptors as well as inhibition of PI3K and PI3K related kinases [23, 42, 43]. The inability of a ryanodine receptor agonist to mimic the effect of caffeine suggests this mechanism is not involved. Caffeine can inhibit all four isoforms of the class I PI3Ks but the potency of this inhibition varies considerably. IC50 data [23] suggests that at the caffeine concentration used in this study (100–200 μM) the inhibitory effect on p110δ (IC50 = 75 μM) would be considerably stronger than its effects on the other isoforms (IC50 ≥ 400 μM). The observation that both caffeine and the selective PI3Kδ inhibitor Idelalisib inhibited both the PI3K activity and suppression associated with activated CLL cells also suggests p110δ inhibition provides the predominant effect. Nonetheless the possibility that the numerous other biological effects of caffeine may also play a role cannot be discounted.

It is likely that caffeine, even at the low levels used in this study, would have additional effects on CLL cells in different immunological contexts. Its activity as an ADR antagonist may inhibit the function of any mobilised Treg and Breg populations and may also inhibit the autocrine effect adenosine has been reported to have in supporting CLL survival [17, 18]. In addition its inhibitory effects on kinases involved in the DNA damage response [43, 44] may modify responses to therapies. A major issue with *in vitro* studies using caffeine is whether the concentrations utilised are biologically relevant [24, 45]. In the current study a significant effect was observed at levels down to 100 μM which is consistent with the IC50 (75 μM) reported for p110δ inhibition [23] and considerably lower than the plasma levels considered to be toxic (400 μM) [46, 47]. A recent study has reported human hemopoietic cells actively take up caffeine and that, in acute myeloid leukemia cells, caffeine inhibited signalling downstream from PI3K at concentrations as low as 100 μM [48]. Although individuals vary markedly with respect to caffeine metabolism, studies to date suggest regular daily consumption of 4–5 cups of coffee or caffeinated energy drinks would result in plasma levels in the region of 100 μM [24, 49, 50]. Given the widespread consumption of varying doses of caffeine the possibility that patient caffeine levels may modulate the effectiveness of other cancer therapies merits further investigation.

The importance of the BCR in CLL pathogenesis has made its downstream signalling pathways, including PI3K, a therapeutic target. Inhibition of PI3Kδ with Idelalisib has proven to be a highly effective therapy [19–21]. This is thought to arise primarily by inhibiting pro survival signals in CLL cells and promoting CLL egress from the protective TME [51, 52] The data in the current study suggests that an additional effect of Idelalisib may be inhibition of the immunosuppressive capacity of activated CLL cells such as those in the TME. As PI3Kδ is widely expressed in leucocytes it is unknown whether this results from effects on the CLL cells, T cells, non-T cells or a combination of these. In agreement with other reports [53] neither caffeine nor Idelalisib affected the proliferation of PBMC cultured alone. It has been reported that broad PI3K inhibition can increase the susceptibility of T cells to suppression by cells such as Tregs [54]. However in the current study PI3Kδ inhibition reduced T cell suppression suggesting it has either no effect, or the opposite effect on susceptibility, possibly reflecting differences in the inhibitor specificity and assay system used. It is possible that the CLL cells do not directly suppress T cells but act indirectly via activation of suppressor populations such as Treg and MDSC within the responder PBMC. However the low numbers of Treg present in PBMC and the inability of Tregs to suppress in proliferation assays with strong CD3/CD28 signalling, such as used in this study, makes involvement of Tregs unlikely [55, 56]. Similarly, the observation that suppression was still observed using CD14 depleted PBMC responders precludes involvement of myeloid derived suppressor cells. The observation that delayed addition of CLL cells does not reduce suppression also suggests that CLL driven expansion of Treg and MDSC populations is not occurring.

Because PI3Kδ is involved in the regulation of multiple signaling pathways in a range of immune cell types it remains unclear how PI3Kδ inhibition results in reduced suppression [57]. It will be important to determine whether it modulates a specific known or novel receptor-ligand/cytokine interaction or induces a more global change in cell function.

Although both caffeine and idelalisib significantly reduced the suppressive effect of activated CLL cells it is clear that this was a partial rather than full reduction. The reasons for this are unclear. It may reflect the presence of multiple rather than a single suppressive mechanism. Additionally as caffeine and Idelalisib predominantly target only one of the PI3K isotypes it may reflect the presence of resistance mechanisms such as feedback loops and compensatory signaling which have been described in the literature [57]. The incomplete reversal of suppressor cell activity using single inhibitors is often observed in *in vitro* assays and may reflect the limitations of such artificial systems. It will be important to analyse the effect of activated CLL cells and PI3Kδ inhibition in the context of other types of *in vitro* and *in vivo* assays in order to determine the full immunological impact of both the suppression and its inhibition.

The concentration of Idelalisib used in this study (0.1 uM) was substantially lower than even the trough levels observed in CLL patients receiving standard doses (1 uM) [19]. Although this concentration was sufficient to inhibit both PI3Kδ and CLL mediated suppression, as expected from other reports, it was not high enough to affect the survival of the CLL cells [51, 58].

It is clear from studies using activated B cells and Bregs prepared from normal donors [13, 15, 38, 59, 60], that immunosuppressive capacity is not a unique characteristic of leukemic B cells. The possibility that the immunosuppressive mechanisms reportedly utilized by normal B cell populations may also be used by activated CLL cells provided the rationale for a number of the experimental approaches used in this study. The finding that adenosine and IL-10 release does not appear to be involved in activated CLL mediated suppression raises the possibility that normal and leukemic B cells may not share the same suppressive mechanisms. This possibility and the impact of PI3Kδ inhibitors on Breg function merit further investigation. Taken together these data demonstrate that both caffeine and the PI3Kδ specific inhibitor Idelalisib inhibit the suppressive activity of activated CLL cells. This previously undescribed activity raises a number of possibilities concerning the use of these compounds. It is thought that targeting the immunosuppressive mechanisms operating within the TME in which activated CLL cells are found is crucial for improving therapeutic outcomes [2, 61]. The addition of caffeine or Idelalisib as an adjunct to therapies targeting other suppressive mechanisms in the TME may therefore be of benefit. The observation that Idelalisib was effective in this role at relatively low doses may also mitigate some of the side effects associated with its current usage. The use of Idelalisib, caffeine or related compounds for the blockade of at least some aspects of CLL mediated immuno-suppression merits further investigation.

## Supporting information

S1 Fig Gating strategy used for determination of relative proliferation. Flow cytometric analysis of CFSE^**+**^PBMC activated 72h with CD3+CD28 in the absence or presence of activated CLL. Cells were labelled with CD3-PE prior to analysis. Plots show (A) forward versus side scatter plots used to set a gate (R1) for viable cells. (B) Plot of CFSE versus CD3-PE fluorescence obtained following gating on R1 was used to identify CD3+ T cells.(C) A histogram of the CFSE fluorescence associated with the gated CD3+T cells. The percentage of T cells undergoing ≥ 3 divisions was quantitated. Percentages were normalised relative to the percentage observed in cultures containing PBMC and CLL cells alone, which represents baseline suppression. These normalised values were defined as relative proliferation (Relative proliferation (%) = percentage ≥ 3 divisions / percentage ≥ 3 divisions in PBMC + CLL cultures x 100).
(TIF)

S2 Fig Effect of caffeine and Idelalisib on PBMC proliferation. CFSE labelled responders were cultured with CD3/CD28 in the presence or absence of Idelalisib or caffeine prior to analysis of proliferation. All data were normalised relative to cultures containing PBMC alone (defined as 100%) and shown as mean ± SEM.
(TIF)
