# Supplementary figures and images for: Idelalisib and caffeine reduce suppression of T cell responses mediated by activated chronic lymphocytic leukemia cells
Source: PLoS One. 2017 Mar 3;12(3):e0172858. doi: 10.1371/journal.pone.0172858 (PMC5336221; doi:10.1371/journal.pone.0172858)

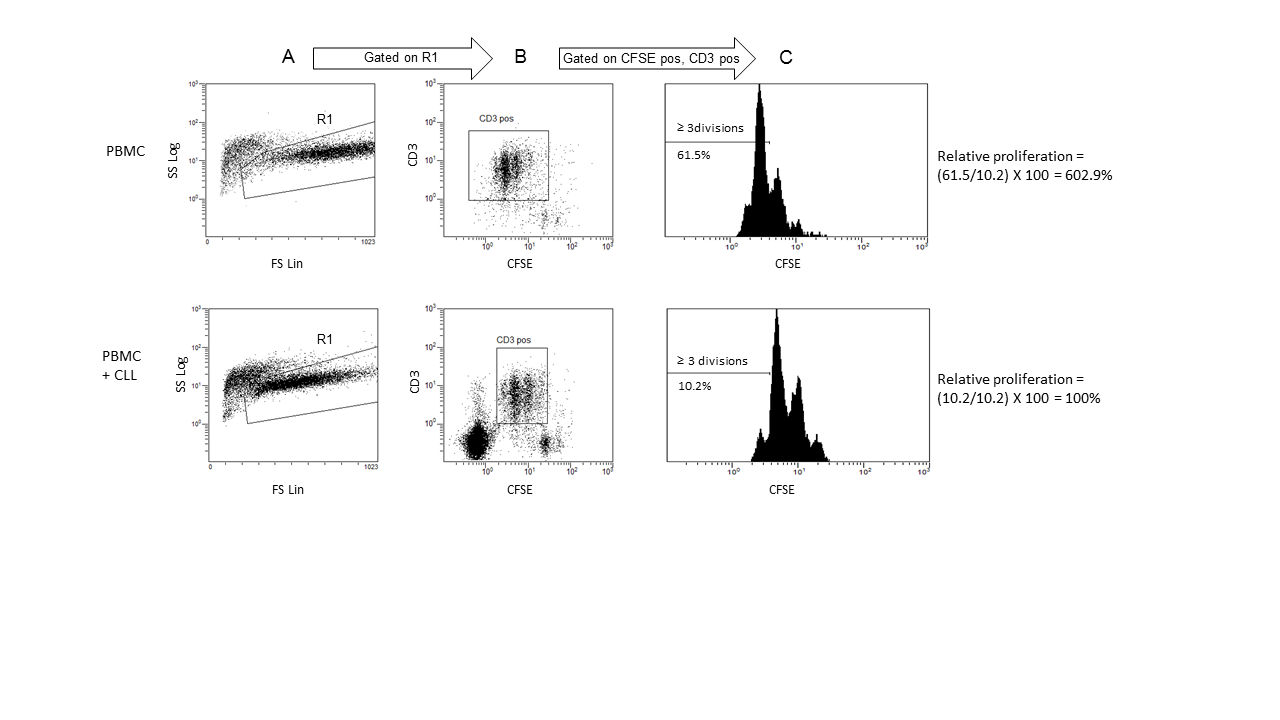

Supplement: S1 Fig — Flow cytometric analysis of CFSE+PBMC activated 72h with CD3+CD28 in the absence or presence of activated CLL. Cells were labelled with CD3-PE prior to analysis. Plots show (A) forward versus side scatter plots used to set a gate (R1) for viable cells. (B) Plot of CFSE versus CD3-PE fluorescence obtained following gating on R1 was used to identify CD3+ T cells.(C) A histogram of the CFSE fluorescence associated with the gated CD3+T cells. The percentage of T cells undergoing ≥ 3 divisions was quantitated. Percentages were normalised relative to the percentage observed in cultures containing PBMC and CLL cells alone, which represents baseline suppression. These normalised values were defined as relative proliferation (Relative proliferation (%) = percentage ≥ 3 divisions / percentage ≥ 3 divisions in PBMC + CLL cultures x 100). (TIF) [file pone.0172858.s001.tif]
